# The influence of physical activity on circadian syndrome: a nationwide prospective study based on the CHARLS cohort

**DOI:** 10.1186/s12889-026-27627-3

**Published:** 2026-05-02

**Authors:** Datong Li, Mian Sun, Wanyu Jiang

**Affiliations:** 1 Graduate School, Emilio Aguinaldo College, Manila, Philippines; 2 Department of Military Training, Officers College of Chinese People’s Armed Police Force, Chengdu, China; 3 College of Primary Education, Huaihua Normal College, Huaihua, China

**Keywords:** Circadian syndrome, Physical activity, Cox regression model, CHARLS, Longitudinal study

## Abstract

**Background:**

A major public health concern is the increasing incidence of circadian syndrome (CircS), especially in older people. While physical activity (PA) is recognized for its metabolic benefits, its longitudinal association with CircS, especially in the Chinese context, remains to be fully elucidated. This research sought to investigate this relationship using a national longitudinal cohort.

**Methods:**

This prospective study utilized data from the China Health and Retirement Longitudinal Study (CHARLS) 2011–2015, including 4,045 participants aged above 45. Participants were categorized as sedentary (PA-), or into low, moderate, and high activity groups based on tertiles. Cox proportional hazards models were established to estimate association between PA and CircS, with adjustments for covariates. Cumulative incidence curves were plotted via the Kaplan-Meier (KM) method. The dose-response relationship was examined with restricted cubic splines (RCS), and subgroup analyses were implemented via stratified Cox regression.

**Results:**

Among the included population (CircS: *n* = 694), a significant, inverse dose-response association was observed between PA levels and the risk of CircS. Compared to the sedentary group, the moderate and the high PA groups were both notably associated with reduced risk of CircS, as reflected by hazard ratios less than 1. KM analysis showed a clear gradient of decreasing CircS incidence with increasing levels of PA. RCS analysis confirmed a linear inverse association. Subgroup analysis showed that the inverse association was consistent across populations defined by aged less than 60 years, male, and other characteristics.

**Conclusion:**

Higher PA levels are linked to a lower risk of developing CircS in middle-aged and older Chinese adults, supporting the potential role of PA in the primary prevention of CircS.

**Trial registration:**

Not applicable.

**Supplementary Information:**

The online version contains supplementary material available at 10.1186/s12889-026-27627-3.

## Introduction

Circadian rhythms are regulated by the coordinated activity of a central pacemaker in the brain and peripheral oscillators in organs throughout the body. These rhythms are driven by transcription factors that regulate cyclic gene expression and influence key physiological processes such as metabolism [1]. Circadian rhythm disruptions are not only associated with sleep disorders, depression, and non-alcoholic fatty liver disease (NAFLD), but also represent significant contributing factors to type 2 diabetes, cardiovascular diseases, and hypertension [2, 3]. Circadian disruption adversely affects cognitive domains including attention, alertness, executive function, and memory [4], which is particularly relevant in middle-aged and older adults, a population with a higher prevalence of circadian disturbances [5]. Circadian syndrome (CircS) is an integrative clinical concept, evolved from metabolic syndrome (MetS), which encompasses cardiometabolic risk factors, depression, sleep disorders, and NAFLD [6, 7]. At least four of the following elements must be present for CircS to be diagnosed: hypertension, dyslipidemia, increased waist circumference, diabetes, insufficient sleep, and depressive symptoms [8–10]. According to studies, CircS dramatically raises the risk of type 2 diabetes, cardiovascular disease, functional disability, periodontitis, and dementia [11–13]. Cross-sectional studies have suggested that CircS is a stronger predictor of cardiovascular disease and psoriasis than MetS alone [14, 15]. Despite these adverse outcomes, current evidence on CircS remains predominantly cross-sectional, and longitudinal studies exploring its modifiable risk factors are still limited. These findings underscore the importance of advancing our understanding of CircS’s impact on older adults’ health, and highlight the urgent need to identify its risk factors and develop targeted interventions to alleviate its multifaceted health consequences.

Physical activity (PA) refers to any movement generated by the contraction of skeletal muscles that leads to the consumption of energy [16]. Evidence-based public health guidelines, such as the second edition of the Physical Activity Guidelines for Americans, recommend the amount, intensity, and types of PA needed for health benefits, including regular moderate-to-vigorous aerobic exercise along with muscle-strengthening activities [17]. PA is now widely recognized as an effective intervention for promoting psychological well-being and preventing mental disorders [18]. It has demonstrated efficacy in alleviating depression and anxiety [19], potentially through mechanisms such as elevating serotonin levels in the brain and enhancing immune function. Furthermore, PA significantly improves sleep quality across different age groups. In addition to its benefits for mental well-being and sleep quality, PA is strongly linked to a lower risk of cardiometabolic abnormalities—core components of CircS (e.g., hypertension, dyslipidemia, diabetes) [20, 21]. Notably, it is worth noting that there have been review studies on the impact of PA on the circadian rhythm system [22], but these research results lack longitudinal validation to confirm the causal relationship. Moreover, few studies have explored whether different intensities or durations of PA have different protective effects on the CircS, which makes the dose-response pattern unclear.

Given the established role of PA in regulating metabolic, mental, and sleep-related health—all core components of CircS—it is plausible that PA exerts protective effects against CircS by stabilizing circadian rhythms. There is still little longitudinal data on this interaction, especially the dose-response relationship between the incidence of CircS and PA. Thus, utilizing data from the China Health and Retirement Longitudinal Study (CHARLS), this study intends to prospectively examine the relationship between varying levels of PA and the risk of developing CircS in a middle-aged and older Chinese population.

## Materials and methodology

### Study design and population

A nationwide longitudinal survey, the CHARLS, focuses on Chinese households and individuals aged 45 and older. Its objective is to gather high-quality microdata to support multidisciplinary research on population aging (approved by the Biomedical Ethics Review Committee of Peking University, IRB00001052-11015). In 2011, a multi-stage probability-proportional-to-size (PPS) sampling technique was employed to conduct the national baseline survey. It included more than 10,000 households and over 17,000 individuals across 450 towns and communities in 150 counties spanning 28 provinces. Every two to three years, the survey is carried out, and participants’ households are used for follow-up waves of computer-assisted personal interviews (CAPI). In addition to 13 physical measurements and blood sample collection, the survey collects a wide range of information, such as basic demographic statistics, intra-household transfers, health status, healthcare and insurance, employment, income, expenses, and assets.

**Fig. 1:**
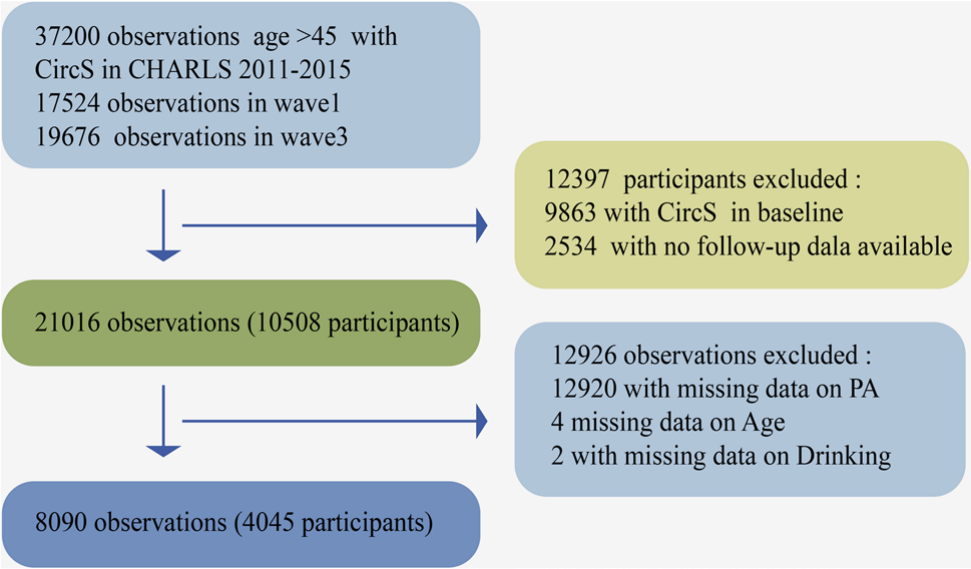
Flowchart showing the selection of the studied population

Based on data from the CHARLS 2011–2015 waves, this study initially included 37,200 observations from participants aged over 45 years, comprising 17,524 observations from Wave 1 (2011) and 19,676 observations from Wave 3 (2015). After applying exclusion criteria, 9,863 participants with CircS at baseline and 2,534 participants lacking follow-up data were removed. An additional 12,926 observations were excluded during data cleaning due to missing data on PA (12,920 observations), age (4 observations), and drinking status (2 observations), leaving a final analytic sample of 4,045 individuals (8,090 observations) (Fig. 1).

### Measurement of PA

Participants’ PA was assessed through a self-reported questionnaire that collected data on the intensity (vigorous, moderate, or walking), frequency (1–7 days per week), and duration (categorized as < 30 min, 30 min–2 h, 2–4 h, or ≥ 4 h (assigned as 4 h)) of weekly activities [23]. The duration for each activity was numerically represented using the midpoint of its corresponding category. The overall weekly PA level, expressed in MET-minutes, was calculated by summing the products of intensity-specific MET values, duration, and frequency, following the standard International Physical Activity Questionnaire (IPAQ) scoring protocol [24].

$$Walking\;MET\;(min/week)=3.3\times walking\;minutes\times walking\;days$$ 

$$\begin{aligned} &Moderate\;MET\;(min/week)=4\times moderate\\&-intensity\;activity\;minutes\times moderate-intensity \end{aligned}$$ 

$$\begin{aligned} &Vigorous\;MET\;(min/week)=8\times vigorous\\&-intensity\;activity\;minutes\times vigorous-intensity\;days \end{aligned}$$ 

$$\begin{aligned} &Overall\;MET\;(min/week)=Walking\;MET\\&+Moderate\;MET+Vigorous\;MET \end{aligned}$$ 

Participants were categorized as either sedentary (PA-, reporting 0 PA, MET=0) or into low (0 < MET<3,000), moderate (3,000 ≤ MET<15,000), or high (MET≥15,000) activity groups based on tertile distributions of their PA values.

### Definition of CircS

If a participant satisfied four or more of the following requirements, they were diagnosed with CircS: increased waist circumference (≥ 85 cm for male, ≥ 80 cm for female); elevated blood pressure (average of three measurements: SBP ≥ 130 mmHg and/or DBP ≥ 85 mmHg) or antihypertensive medication use; low HDL-C (< 40 mg/dL for male, < 50 mg/dL for female) or lipid-lowering medication use; hypertriglyceridemia (≥ 150 mg/dL) or lipid-lowering medication use; elevated fasting glucose (≥ 100 mg/dL) or glucose-lowering medication use; short sleep duration (total sleep time from self-report < 6 h/day); and symptoms of depression (Center for Epidemiologic Studies Depression Scale [CESD-10] score ≥ 10) [25].

### Covariates

The following covariates were included in the analysis: age (continuous variable, years), gender (male, female), education level (primary education or lower, secondary school and above), marital status (married, others), residence (urban, rural), smoking status (yes, no), drinking status (yes, no).

### Statistical analysis

The normality of age, a continuous variable [interquartile range, IQR], was evaluated using the Shapiro-Wilk test, and was compared across groups with the Kruskal-Wallis H test. Other categorical variables (residence, marital status, gender, smoking status, educational attainment, and drinking status), expressed as frequencies (percentages), were compared across groups via the chi-square test.

Cox proportional hazards regression models were employed in longitudinal analyses to assess the relationship between PA and CircS. The censoring scheme was defined as follows: a new onset of CircS during the follow-up period was defined as an event (status = 1). The event was defined as censored (status = 0) if any of the following conditions were met: (1) no onset occurred during the follow-up period until the study endpoint; (2) loss to follow-up; (3) death due to any cause before the onset. The proportional hazards assumption of the model was also tested using the Schoenfeld residuals method. The “time to onset” of CircS was defined as the time from the baseline survey point to the first year in the follow-up survey when it is identified as meeting the diagnostic criteria for CircS. The difference in years is calculated to obtain the onset time in “years”. Three sequentially adjusted models were established via the survey (v4.2-2) package [26]: Model 1 was a crude, unadjusted model; Model 2 incorporated age, residence, gender, educational attainment, and marital status; and Model 3 built upon Model 2 by additionally accounting for smoking and drinking. The receiver operator characteristic (ROC) curve was constructed to assess the performance of the model. Cumulative incidence curves were plotted using the Kaplan-Meier (KM) method with the survminer (v0.5.0) package [27]. To explore the dose-response relationship, a restricted cubic spline (RCS) model was fitted to examine potential non-linearity, with the likelihood ratio test utilized to assess the statistical significance of non-linear trends. Subgroup analyses were conducted based on Model 3 to examine the potential effects of PA on CircS modifications according to participant characteristics adopting stratified Cox regression.

Sensitivity analyses were undertaken to verify the robustness of primary results across diverse dimensions. First, we conducted a sensitivity analysis using logistic regression based on the 2015 cross-sectional data of CHARLS to confirm the robustness of the core findings. Second, to eliminate the influence of the grouping method on the results, we conducted a sensitivity analysis by re-grouping using the sample quartiles. The groups were sedentary (MET = 0), low (0 < MET < 4158), moderate (4158 ≤ MET < 11812), and high (MET ≥ 11812). Third, we have combined the sedentary and low PA groups into a new low-activity group and re-performed the sensitivity analysis. Furthermore, we constructed a nomogram to visualize the risk prediction model. Model calibration was assessed using the bootstrap resampling method with the pec package (v2025.6.24) to evaluate the agreement between predicted probabilities and observed risks. R (v4.4.3) was applied for all statistical analyses, and two-tailed *P*-value of less than 0.05 was regarded as statistically significant.

## Results

### Baseline characteristics

The baseline analysis included 4,045 participants with a median age of 57 years (IQR: 50.0–64.0) and a relatively balanced sex distribution (51% female and 49% male) (Table 1). When participants were categorized by CircS, notable differences emerged between the CircS group (*n* = 694) and the normal group (*n* = 3,351) (*P* < 0.01). PA levels differed significantly between the two groups (*P* = 0.007). The CircS group had higher proportions of participants with no PA (12%) and low PA (25%), but lower proportions of moderate PA (43%) and high PA (19%) compared with the normal group. Gender composition, residential location, smoking status, and drinking status all differed significantly between the CircS and normal groups (all *P* < 0.001). Specifically, the CircS group had a higher proportion of females (61% vs. 49%) and people living in urban (41% vs. 34%), but lower rates of smoking (25% vs. 34%) and drinking (28% vs. 36%).

**Table 1:** Baseline characteristics of participants from CHARLS 2011

Variable	Overall	CircS	Normal	*P*
	4045	694	3,351	
PA				0.007
PA-	384 (9%)	83 (12%)	301 (9%)	
Low	937 (23%)	175 (25%)	762 (23%)	
Moderate	1,787 (44%)	301 (43%)	1,486 (44%)	
High	937 (23%)	135 (19%)	802 (24%)	
Age				0.991
Median (Q1, Q3)	57.0 (50.0, 64.0)	57.0 (50.0, 64.0)	57.0 (50.0, 64.0)	
Gender				< 0.001
Female	2,051 (51%)	424 (61%)	1,627 (49%)	
Male	1,994 (49%)	270 (39%)	1,724 (51%)	
Education				0.858
Primary education or lower	2,754 (68%)	475 (68%)	2,279 (68%)	
Secondary school and above	1,291 (32%)	219 (32%)	1,072 (32%)	
Marry				0.358
Married	3,624 (90%)	629 (91%)	2,995 (89%)	
Other	421 (10%)	65 (9%)	356 (11%)	
Residence				< 0.001
Urban	1,428 (35%)	284 (41%)	1,144 (34%)	
Rural	2,617 (65%)	410 (59%)	2,207 (66%)	
Smoking				< 0.001
NO	2,742 (68%)	521 (75%)	2,221 (66%)	
YES	1,303 (32%)	173 (25%)	1,130 (34%)	
Drinking				< 0.001
NO	2,645 (65%)	501 (72%)	2,144 (64%)	
YES	1,400 (35%)	193 (28%)	1,207 (36%)	

*PA* Physical activity

### The inverse association between PA and CircS

Based on the results of the Cox regression analysis, higher levels of PA showed a notable inverse association with the risk of CircS in a dose-response manner (*P* < 0.05) (Table 2). In Model 1, compared to the sedentary group (PA-), moderate and high PA levels were associated with a notably lower risk of CircS, with hazard ratios (HRs) and 95% CI of 0.753 (0.591–0.960) and 0.622 (0.473–0.818), respectively. After adjusting in Model 2, the results remained consistent with HRs being 0.778 (0.609–0.994) for moderate activity and 0.697 (0.527–0.922) for high activity. In Model 3, the associations were slightly attenuated but remained statistically significant for moderate (HR = 0.780, 0.611–0.997) and high PA (HR = 0.703, 0.532–0.931). These findings suggested that moderate and high levels of PA were associated with a reduced risk of CircS. The Schoenfeld residual test revealed that the core variable PA satisfies the proportional hazards assumption (Table S1).

**Table 2:** Cox regression models examining association of PA and CircS

PA	Model 1		Model 2		Model3	
	HR(95% CI)	*P*	HR(95% CI)	*P*	HR(95% CI)	*P*
PA-	Ref.		Ref.		Ref.	
Low	0.856 (0.659–1.112)	0.244	0.842 (0.647–1.094)	0.198	0.837 (0.644–1.089)	0.185
Moderate	0.753 (0.591–0.960)	0.022	0.778 (0.609–0.994)	0.044	0.780 (0.611–0.997)	0.047
High	0.622 (0.473–0.818)	< 0.001	0.697 (0.527–0.922)	0.011	0.703 (0.532–0.931)	0.014

*PA* Physical activity, *HR* Hazard ratios, *CI* Confidence interval

**Fig. 2:**
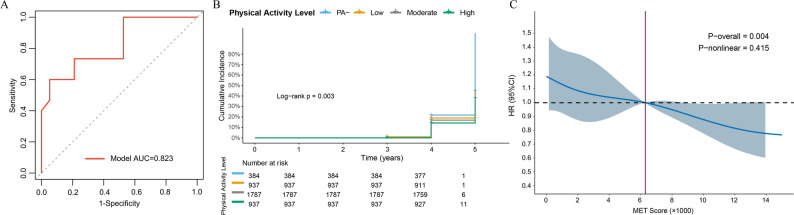
The relationship between physical activity (PA) and circadian syndrome (CircS). (**A**) Receiver operator characteristic (ROC) curve constructed to assess the performance of the model (**B**) Cumulative incidence curves plotting to visualize the incidence of CircS stratified by physical activity (PA) levels. (**C**) Restricted cubic spline (RCS) fitted to examine potential non-linearity

Model 3 exhibited robust predictive capability, achieving an area under the curve (AUC) value of 0.823 (Fig. 2A). KM analysis showed a clear gradient of decreasing CircS incidence with increasing levels of PA (*P* = 0.003). The high-activity group consistently demonstrated the lowest incidence of CircS across the follow-up period, supporting the premise of a persistent and cumulative underlying inverse association (Fig. 2B). The RCS analysis revealed an inverse linear relationship between PA levels and the risk of CircS (*P*-overall = 0.004, and *P*-non-linear = 0.415) (Fig. 2C).

### The inverse associations of PA with CircS remained in population strata

**Fig. 3:**
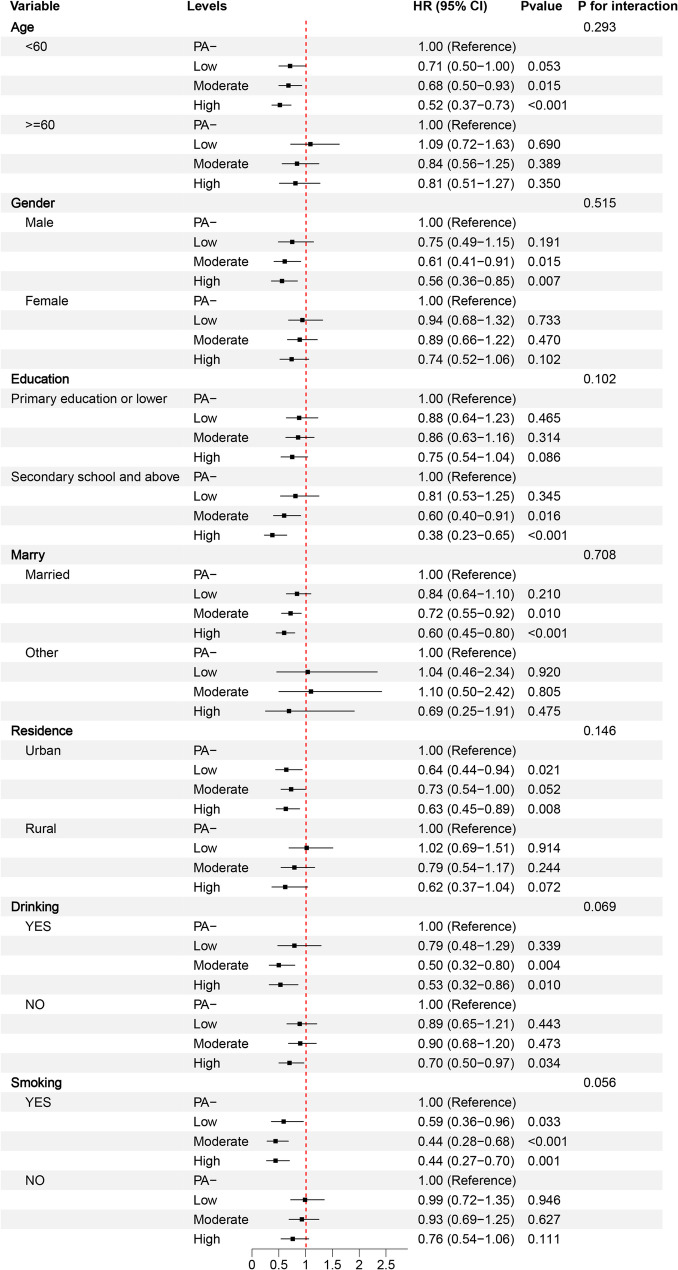
The relationship between PA and CircS in various populations

Based on the subgroup analyses, the inverse associations of PA with CircS were consistently observed across various population strata (*P* < 0.05) (Fig. 3). Notably, significant inverse associations were evident among participants aged < 60 years (moderate PA: HR = 0.68, 0.50–0.93; high PA: HR = 0.52, 0.37–0.73), males (moderate PA: HR = 0.61, 0.41–0.91; high PA: HR = 0.56, 0.36–0.85), those with secondary school education and above (moderate PA: HR = 0.60, 0.40–0.91; high PA: HR = 0.38, 0.23–0.65), married individuals (moderate PA: HR = 0.72, 0.55–0.92; high PA: HR = 0.60, 0.45–0.80), drinkers (moderate PA: HR = 0.50, 0.32–0.80; high PA: HR = 0.53, 0.32–0.86), and smokers (low PA: HR = 0.59, 0.36–0.96; moderate PA: HR = 0.44, 0.28–0.68; high PA: HR = 0.44, 0.27–0.70).

### Sensitivity analysis

To verify the robustness of the results, we conducted a sensitivity analysis using logistic regression based on the 2015 cross-sectional data of CHARLS. The results showed that in the fully adjusted model, moderate and high PA levels were still significantly associated with a reduced risk of CircS (moderate PA: odds ratio (OR) = 0.812; high PA: OR = 0.629), while the association of low-intensity PA was not statistically significant (Table S2). Meanwhile, sensitivity analysis using exposure levels reclassified by tertiles yielded results highly consistent with those from the Cox regression in the main analysis (Table S3). After combining the sedentary group with the low PA group to form a new low-activity group, a sensitivity analysis was conducted again. Compared with the low-activity group, moderate and high levels of PA were still associated with a reduced risk of CircS. Additionally, in model 3, the inverse effect of the high-activity group remained robust (HR = 0.793, 95% CI: 0.636–0.989, *P* = 0.040) (Table S4). In summary, above sensitivity analyses supported the robustness of the negative association between PA and the risk of CircS.

### Prediction the risk of impaired CircS based on clinical variables

To further verify the clinical predictive value of PA in relation to the occurrence of CircS, we constructed a nomogram based on all the included variables and conducted a comprehensive assessment of its predictive performance (Figure S1A). The time-dependent ROC curve analysis revealed that the AUC value of the model in the fourth year of follow-up was 0.823, indicating that the model had a good ability to distinguish the risk of CircS occurrence. However, the calibration curve showed that the predicted risk values by the model were systematically higher than the actual observed risk values, suggesting a mild overestimation tendency (Figure S1B). Overall, although this nomogram model demonstrates certain risk discrimination efficacy, there is still room for improvement in its calibration performance. The predictive results should be interpreted with caution in combination with actual circumstances.

## Discussion

PA levels and the likelihood of developing CircS were found to be markedly inversely correlated in this large, prospective cohort study of middle-aged and older Chinese adults. The results demonstrated a clear dose-response relationship, wherein increasing PA levels were associated with progressively lower hazards of CircS. Specifically, even after thorough correction for demographic and lifestyle factors, those who engaged in moderate and high levels of PA showed a significantly lower risk than their sedentary peers. This inverse association was remarkably consistent across various population subgroups, and the dose-response pattern was further verified as linear by RCS analysis. These robust findings collectively suggested that regular PA, particularly at moderate to high intensities, may confer significant protection against the development of CircS.

The inverse association between PA and the risk of CircS observed in our study aligns closely with the well-documented benefits of PA on metabolic health and cardiovascular diseases reported in existing literature. PA elevates energy requirements in various tissues and triggers both immediate and long-term adjustments in metabolic processes [28]. Substantial evidence demonstrates that higher levels of PA are consistently associated with improved metabolic parameters. Metabolomic studies reveal that exercise induces characteristic changes, including increases in pyruvate, lactate, acylcarnitines, fatty acids, TCA cycle intermediates, and ketone bodies, while decreasing bile acids within the first 24 h post-exercise [29]. Regular PA is linked to significant improvements in systolic blood pressure (mean effect: -3 mmHg), diastolic blood pressure (mean effect: -3 mmHg), body weight (reduction of 2–3 kg), and HDL-C levels (increase of 1–2 mg/dL), collectively contributing to a 21% overall reduction in CVD risk [30]. These metabolic adaptations are reflected in population studies showing increased PA is inversely associated with biomarkers for alanine, acetoacetate, VLDL, lactate, LDL, and the inflammatory marker glycoprotein acetylation—all factors strongly linked to cardiovascular disease risk [31]. Notably, an inverse correlation exists between PA and the prevalence of both hypertension and dyslipidemia, as individuals meeting aerobic, muscle-strengthening, or combined guidelines show 12–33% and 6–24% lower odds of these conditions, respectively [32, 33]. The enhanced carbon fuel consumption due to increased TCA cycle activity underscores the role of sustained aerobic training in improving VO₂ max and connecting TCA cycle efficiency to cardiorespiratory fitness [28]. Large-scale epidemiological studies further indicate that adults with higher body mass index tend to exhibit lower levels of moderate-to-vigorous PA (MVPA), highlighting the interconnected nature of metabolic health and PA patterns [33]. As a fundamental component of ideal cardiovascular health (CVH), PA is widely recommended for both prevention and management of cardiovascular disease (CVD) [34]. Robust evidence confirms that MVPA exerts beneficial effects on all-cause mortality, CVD mortality, and atherosclerotic CVD—including incident coronary heart disease, ischemic stroke, and heart failure [35, 36]. These established cardiometabolic benefits, supported by both molecular and epidemiological evidence, provide a comprehensive mechanistic foundation for understanding the inverse association between PA and CircS observed in our investigation.

A growing body of evidence demonstrates the beneficial effects of PA on mental health. Exercise has been shown to enhance mood and self-esteem while reducing stress susceptibility [37]. Notably, exercise and physical training may improve depressive symptoms with efficacy comparable to or even exceeding that of conventional antidepressants [38]. Multiple neurobiological mechanisms underlie these psychological benefits. According to the “endorphin hypothesis,” PA increases brain production of endogenous opioids, thereby reducing pain perception and improving mood [39]. Additionally, exercise-induced endocannabinoids are associated with pleasure, anxiolytic effects, and reduced pain sensitivity [40]. Regular PA also optimizes hypothalamic-pituitary-adrenal (HPA) axis function, reducing cortisol secretion and restoring balance to leptin and ghrelin levels [41]. The neurological benefits of exercise are mediated through multiple mechanisms, including enhanced neuroplasticity, reduced neuroinflammation, and improved metabolic and neurotransmitter homeostasis [42, 43]. PA mediates its positive effects on neuropsychiatric conditions, including the alleviation of depressive symptoms, through the upregulation of key exerkines such as irisin, adiponectin, and brain-derived neurotrophic factor (BDNF) [44]. Exercise also exerts immunomodulatory effects, including optimized catecholamine levels, reduced cortisol, and decreased systemic inflammation. These adaptations are particularly relevant given that sleep deprivation negatively impacts immune function, mood, glucose metabolism, and cognition [45]. These established mechanisms provide a scientific basis for understanding how PA may target specific components of CircS. The mood-enhancing effects of PA directly address the depressive symptoms incorporated in CircS diagnosis, while its sleep-promoting and immune-regulating functions counter the sleep disturbances and metabolic dysregulation characteristic of this syndrome. The coordinated improvement across multiple physiological systems—neural, endocrine, and immune—suggests that regular PA represents a comprehensive intervention strategy capable of simultaneously addressing the interconnected components of CircS. This mechanistic understanding supports our epidemiological findings of an inverse association between PA levels and CircS risk, suggesting that the psychological and neurological benefits of exercise contribute significantly to its observed inverse association against this integrated clinical condition.

This study possesses several notable strengths, including its nationally representative, prospective cohort design with a large sample size, which provides high-quality epidemiological evidence. To our knowledge, this is the first investigation to systematically examine the association between PA and CircS in a middle-aged and older Chinese population. The robustness of our findings is reinforced by the use of multivariable adjustment, dose-response analysis, and subgroup analyses. But there are certain limitations to take into account as well. First, PA was self-reported, which makes it prone to misclassification and measurement error. Second, residual confounding from unmeasured factors cannot be completely ruled out, even though we corrected for a wide variety of possible confounders. Third, our results cannot prove causation because the study was observational. Fourth, considering that the study population was composed of middle-aged and older persons, care should be taken when extrapolating the findings to other age groups. Fifth, the CircS status exhibits dynamic fluctuations, and the discrete follow-up results in interval censored data. The Cox model only focuses on the time of the first onset and is unable to fully reflect the characteristics of the disease. Although we conducted sensitivity analyses to verify the results, we still cannot completely eliminate the influence of bias. Future studies can increase the follow-up frequency and use models more suitable for interval censored data to verify the results. Sixth, the study population was restricted to Chinese individuals, and thus the generalizability and external validity of the present findings to other ethnic groups or regions may be limited. Further studies involving diverse populations are warranted to verify the general applicability of our results. Finally, in the subgroup analysis, some subgroups did not reach statistical significance, which might be due to the insufficient statistical power caused by the relatively small sample size of the subgroups. However, since the interaction test did not indicate significant heterogeneity of the effects, this limitation is not sufficient to undermine the main conclusion of this study.

## Conclusion

In this large, prospective cohort of middle-aged and older Chinese adults, we found a significant, inverse dose-response relationship between PA levels and the risk of developing CircS. This inverse association remained consistent across various population subgroups defined by age, gender, educational level, and lifestyle factors. This study suggests that PA may be considered in comprehensive chronic disease prevention strategies, particularly for circadian health management in aging populations. Furthermore, these results establish a foundation for future research exploring the biological mechanisms through which PA influences CircS.

## Supplementary Information

Supplementary Material 1

Supplementary Material 2. Figure S1 Construction and validation of the nomogram. (A) The nomogram constructed based on covariates. (B) The calibration curve of the nomogram.

## Data Availability

The data for this study were sourced from CHARLS (https://charls.pku.edu.cn/).

## Abbreviations

NAFLD: Non-alcoholic fatty liver disease
CircS: Circadian syndrome
MetS: Metabolic syndrome
PA: Physical activity
CHARLS: China Health and Retirement Longitudinal Study
PPS: Probability-proportional-to-size
CAPI: Computer-assisted personal interviews
IPAQ: International Physical Activity Questionnaire
IQR: Interquartile range
ROC: Receiver operator characteristic
KM: Kaplan-Meier
RCS: Restricted cubic spline

## References

[1] Asher G, Schibler U. Crosstalk between components of circadian and metabolic cycles in mammals. Cell Metabol. 2011;13(2):125–37. 10.1016/j.cmet.2011.01.006 21284980

[2] Ferraz-Bannitz R, Beraldo RA, Coelho PO, Moreira AC, Castro M, Foss-Freitas MC. Circadian Misalignment Induced by Chronic Night Shift Work Promotes Endoplasmic Reticulum Stress Activation Impacting Directly on Human Metabolism. Biology 2021;10(3):197. 10.3390/biology10030197 PMC7998626 33807589

[3] Crnko S, Du Pré BC, Sluijter JPG, Van Laake LW. Circadian rhythms and the molecular clock in cardiovascular biology and disease. Nat reviews Cardiol. 2019;16(7):437–47. 10.1038/s41569-019-0167-4 30796369

[4] Taillard J, Sagaspe P, Philip P, Bioulac S. Sleep timing, chronotype and social jetlag: Impact on cognitive abilities and psychiatric disorders. Biochem Pharmacol. 2021;191:114438. 33545116 10.1016/j.bcp.2021.114438

[5] Rabinowitz JA, An Y, He L, Alfini AJ, Zipunnikov V, Wu MN, Wanigatunga SK, Schrack JA, Jackson CL, Ferrucci L, et al. Associations of circadian rest/activity rhythms with cognition in middle-aged and older adults: Demographic and genetic interactions. Front Neurosci. 2022;16:952204. 36312032 10.3389/fnins.2022.952204 PMC9597505

[6] Zimmet P, Alberti K, Stern N, Bilu C, El-Osta A, Einat H, Kronfeld-Schor N. The Circadian Syndrome: is the Metabolic Syndrome and much more! J Intern Med. 2019;286(2):181–91. 31081577 10.1111/joim.12924 PMC6851668

[7] Akbar Z, Shi Z. Dietary Patterns and Circadian Syndrome among Adults Attending NHANES 2005–2016. Nutrients 2023;15(15):3396. 10.3390/nu15153396 PMC10421411 37571333

[8] Shi Z, Tuomilehto J, Kronfeld-Schor N, Alberti GK, Stern N, El-Osta A, Bilu C, Einat H, Zimmet P. The circadian syndrome predicts cardiovascular disease better than metabolic syndrome in Chinese adults. J Intern Med. 2021;289(6):851–60. 33340184 10.1111/joim.13204

[9] Sun L, Huo X, Jia S, Chen X. The Association between Circadian Syndrome and Frailty in US adults: a cross-sectional study of NHANES Data from 2007 to 2018. Aging Clin Exp Res. 2024;36(1):105. 38713270 10.1007/s40520-024-02745-3 PMC11076391

[10] Arabi A, Nasrallah D, Mohsen S, Abugharbieh L, Al-Hashimi D, AlMass S, Albasti S, Al-Ajmi SA, Khan MN, Zughaier SM. Association between Serum Vitamin D Status and Circadian Syndrome: A Cross-Sectional Study. Nutrients 2024;16(13):2111. 10.3390/nu16132111 PMC11243086 38999859

[11] Huang Y, Wang K, He Y, Wang W, Hu C, Jiang K, Zhang J, Tao Y, Jin L. Association of circadian syndrome with functional disability among middle-aged and older adults in China. J Nutr Health Aging. 2025;29(9):100621. 40602076 10.1016/j.jnha.2025.100621 PMC12271891

[12] Zhang R, Han C, Hu D, Chen Q, Zheng J, Chen J, Okinaga T. Serum lipids, oxidative stress, and systemic inflammation mediate the association between circadian syndrome and periodontitis. Front Nutr. 2025;12:1622348. 40672423 10.3389/fnut.2025.1622348 PMC12263390

[13] Yu L, Liu W, Liao C, Shen N, Liu A, Cheng L, Wang X. The interaction between circadian syndrome and genetic susceptibility in the risk of incident dementia: A longitudinal cohort study. J Prev Alzheimer’s disease. 2025;12(5):100089. 39922757 10.1016/j.tjpad.2025.100089 PMC12183943

[14] Shi Z, Tuomilehto J, Kronfeld-Schor N, Alberti G, Stern N, El-Osta A, Chai Z, Bilu C, Einat H, Zimmet P. The Circadian Syndrome Is a Significant and Stronger Predictor for Cardiovascular Disease than the Metabolic Syndrome-The NHANES Survey during 2005–2016. *Nutrients* 2022;14(24):5317. 10.3390/nu14245317 PMC9785664 36558476

[15] Gu Y, Ye X, Zhao W, He S, Zhang W, Zeng X. The circadian syndrome is a better predictor for psoriasis than the metabolic syndrome via an explainable machine learning method - the NHANES survey during 2005–2006 and 2009–2014. Front Endocrinol. 2024;15:1379130. 10.3389/fendo.2024.1379130 PMC11233539 38988999

[16] Zhao H, Lu C, Yi C. Physical Activity and Sleep Quality Association in Different Populations: A Meta-Analysis. Int J Environ Res Public Health 2023;20(3):1864. 10.3390/ijerph20031864 PMC9914680 36767229

[17] Katzmarzyk PT, Jakicic JM. Physical Activity for Health-Every Minute Counts. JAMA. 2023;330(3):213–4. 37462698 10.1001/jama.2023.11014

[18] Firth J, Solmi M, Wootton RE, Vancampfort D, Schuch FB, Hoare E, Gilbody S, Torous J, Teasdale SB, Jackson SE, et al. A meta-review of lifestyle psychiatry: the role of exercise, smoking, diet and sleep in the prevention and treatment of mental disorders. World psychiatry: official J World Psychiatric Association (WPA). 2020;19(3):360–80. 10.1002/wps.20773 PMC7491615 32931092

[19] Kayani S, Kiyani T, Kayani S, Morris T, Biasutti M, Wang J. Physical Activity and Anxiety of Chinese University Students: Mediation of Self-System. Int J Environ Res Public Health 2021;18(9):4468. 10.3390/ijerph18094468 PMC8122769 33922351

[20] Haskell WL, Lee IM, Pate RR, Powell KE, Blair SN, Franklin BA, Macera CA, Heath GW, Thompson PD, Bauman A. Physical activity and public health: updated recommendation for adults from the American College of Sports Medicine and the American Heart Association. Med Sci Sports Exerc. 2007;39(8):1423–34. 17762377 10.1249/mss.0b013e3180616b27

[21] Lee IM, Shiroma EJ, Lobelo F, Puska P, Blair SN, Katzmarzyk PT. Effect of physical inactivity on major non-communicable diseases worldwide: an analysis of burden of disease and life expectancy. Lancet. 2012;380(9838):219–29. 22818936 10.1016/S0140-6736(12)61031-9 PMC3645500

[22] Weinert D, Gubin D. The Impact of Physical Activity on the Circadian System: Benefits for Health, Performance and Wellbeing. Appl Sci. 2022;12(18):9220.

[23] Lin D, Zhai S, Cui F, Yang Y, Wang H, Wang J, Wei Y. The effect of daily physical activity on bidirectional transitions of elevated blood pressure status: the first longitudinal evidence from the CHARLS. BMC Public Health. 2025;25(1):2104. 40474154 10.1186/s12889-025-23296-w PMC12139308

[24] Xu X, Xu Y, Shi R. Association between obesity, physical activity, and cognitive decline in Chinese middle and old-aged adults: a mediation analysis. BMC Geriatr. 2024;24(1):54. 38212676 10.1186/s12877-024-04664-4 PMC10785530

[25] Ran J, Tao C, Zhang S, Chen Q, Yang P, Hu Y, Liao X. Circadian syndrome is associated with the development of chronic kidney disease and rapid decline in kidney function in middle-aged and elder adults: a China nationwide cohort study. J Nutr Health Aging. 2024;28(1):100011. 38267153 10.1016/j.jnha.2023.100011 PMC12877766

[26] Liang J, An H, Hu X, Gao Y, Zhou J, Gong X, Zong J, Liu Y. Correlation between chronic kidney disease and all-cause mortality in diabetic foot ulcers: evidence from the 1999–2004 national health and nutrition examination survey (NHANES). Front Endocrinol. 2025;16:1533087. 10.3389/fendo.2025.1533087 PMC11949790 40162314

[27] Shi Y, Wang Y, Dong H, Niu K, Zhang W, Feng K, Yang R, Zhang Y. Crosstalk of ferroptosis regulators and tumor immunity in pancreatic adenocarcinoma: novel perspective to mRNA vaccines and personalized immunotherapy. Apoptosis: Int J Program cell death. 2023;28(9–10):1423–35. 10.1007/s10495-023-01868-8 PMC10425492 37369808

[28] Lim RMH, Koh AS. Cardiovascular Aging and Physical Activity: Insights From Metabolomics. Front Cardiovasc Med. 2021;8:728228. 34616784 10.3389/fcvm.2021.728228 PMC8488139

[29] Schranner D, Kastenmüller G, Schönfelder M, Römisch-Margl W, Wackerhage H. Metabolite Concentration Changes in Humans After a Bout of Exercise: a Systematic Review of Exercise Metabolomics Studies. Sports Med - open. 2020;6(1):11. 32040782 10.1186/s40798-020-0238-4 PMC7010904

[30] Barone Gibbs B, Hivert MF, Jerome GJ, Kraus WE, Rosenkranz SK, Schorr EN, Spartano NL, Lobelo F. Physical Activity as a Critical Component of First-Line Treatment for Elevated Blood Pressure or Cholesterol: Who, What, and How? A Scientific Statement From the American Heart Association. *Hypertension (Dallas, Tex*: 1979) 2021, 78(2):e26-e37. 10.1161/HYP.0000000000000196 34074137

[31] Pang Y, Kartsonaki C, Du H, Millwood IY, Guo Y, Chen Y, Bian Z, Yang L, Walters R, Bragg F, et al. Physical Activity, Sedentary Leisure Time, Circulating Metabolic Markers, and Risk of Major Vascular Diseases. Circulation Genomic precision Med. 2019;12(9):386–96. 10.1161/CIRCGEN.118.002527 PMC6752700 31461308

[32] Omura JD, Hyde ET, Imperatore G, Loustalot F, Murphy L, Puckett M, Watson KB, Carlson SA. Trends in Meeting the Aerobic Physical Activity Guideline Among Adults With and Without Select Chronic Health Conditions, United States, 1998–2018. J Phys Act Health. 2021;18(S1):S53–63. 34465653 10.1123/jpah.2021-0178 PMC10977617

[33] Bennie JA, De Cocker K, Teychenne MJ, Brown WJ, Biddle SJH. The epidemiology of aerobic physical activity and muscle-strengthening activity guideline adherence among 383,928 U.S. adults. Int J Behav Nutr Phys Act. 2019;16(1):34. 30999896 10.1186/s12966-019-0797-2 PMC6472085

[34] Grundy SM, Stone NJ, Bailey AL, Beam C, Birtcher KK, Blumenthal RS, Braun LT, de Ferranti S, Faiella-Tommasino J, Forman DE et al. 2018 AHA/ACC/AACVPR/AAPA/ABC/ACPM/ADA/AGS/APhA/ASPC/NLA/PCNA Guideline on the Management of Blood Cholesterol: A Report of the American College of Cardiology/American Heart Association Task Force on Clinical Practice Guidelines. *Circulation* 2019, 139(25):e1082-e1143. 10.1161/CIR.0000000000000625 PMC7403606 30586774

[35] Kraus WE, Powell KE, Haskell WL, Janz KF, Campbell WW, Jakicic JM, Troiano RP, Sprow K, Torres A, Piercy KL. Physical Activity, All-Cause and Cardiovascular Mortality, and Cardiovascular Disease. Med Sci Sports Exerc. 2019;51(6):1270–81. 31095084 10.1249/MSS.0000000000001939 PMC6527136

[36] Whelton PK, Carey RM, Aronow WS, Casey DE Jr., Collins KJ, Dennison Himmelfarb C, DePalma SM, Gidding S, Jamerson KA, Jones DW et al. 2017 ACC/AHA/AAPA/ABC/ACPM/AGS/APhA/ASH/ASPC/NMA/PCNA Guideline for the Prevention, Detection, Evaluation, and Management of High Blood Pressure in Adults: A Report of the American College of Cardiology/American Heart Association Task Force on Clinical Practice Guidelines. *Circulation* 2018, 138(17):e484-e594. 10.1161/CIR.0000000000000596 30354654

[37] Biddle S. Physical activity and mental health: evidence is growing. World psychiatry: official J World Psychiatric Association (WPA). 2016;15(2):176–7. 10.1002/wps.20331 PMC4911759 27265709

[38] Noetel M, Sanders T, Gallardo-Gómez D, Taylor P, Del Pozo Cruz B, van den Hoek D, Smith JJ, Mahoney J, Spathis J, Moresi M, et al. Effect of exercise for depression: systematic review and network meta-analysis of randomised controlled trials. BMJ (Clinical Res ed). 2024;384:e075847. 10.1136/bmj-2023-075847 PMC10870815 38355154

[39] Mahindru A, Patil P, Agrawal V. Role of Physical Activity on Mental Health and Well-Being: A Review. Cureus. 2023;15(1):e33475. 36756008 10.7759/cureus.33475 PMC9902068

[40] Desai S, Borg B, Cuttler C, Crombie KM, Rabinak CA, Hill MN, Marusak HA. A Systematic Review and Meta-Analysis on the Effects of Exercise on the Endocannabinoid System. Cannabis cannabinoid Res. 2022;7(4):388–408. 34870469 10.1089/can.2021.0113 PMC9418357

[41] Telles S, Gupta RK, Bhardwaj AK, Singh N, Mishra P, Pal DK, Balkrishna A. Increased Mental Well-Being and Reduced State Anxiety in Teachers After Participation in a Residential Yoga Program. Med Sci Monit basic Res. 2018;24:105–12. 30061552 10.12659/MSMBR.909200 PMC6083945

[42] Chen C, Nakagawa S. Physical activity for cognitive health promotion: An overview of the underlying neurobiological mechanisms. Ageing Res Rev. 2023;86:101868. 36736379 10.1016/j.arr.2023.101868

[43] Vancampfort D, Firth J, Stubbs B, Schuch F, Rosenbaum S, Hallgren M, Deenik J, Ward PB, Mugisha J, Van Damme T, et al. The efficacy, mechanisms and implementation of physical activity as an adjunctive treatment in mental disorders: a meta-review of outcomes, neurobiology and key determinants. World psychiatry: official J World Psychiatric Association (WPA). 2025;24(2):227–39. 10.1002/wps.21314 PMC12079350 40371806

[44] Wang N, Zhu S, Chen S, Zou J, Zeng P, Tan S. Neurological mechanism-based analysis of the role and characteristics of physical activity in the improvement of depressive symptoms. Rev Neurosci. 2025;36(5):455–78. 39829004 10.1515/revneuro-2024-0147

[45] Dolezal BA, Neufeld EV, Boland DM, Martin JL, Cooper CB. Interrelationship between Sleep and Exercise: A Systematic Review. Adv Prev Med 2017, 2017:5979510. 10.1155/2017/1364387 PMC5385214 28458924

