# Supplementary material for: The influence of physical activity on circadian syndrome: a nationwide prospective study based on the CHARLS cohort
Source: BMC Public Health. 2026 May 2;26:1909. doi: 10.1186/s12889-026-27627-3 (PMC13281635; doi:10.1186/s12889-026-27627-3)
Supplement: Supplementary file 1 — Supplementary Material 1 [file 12889_2026_27627_MOESM1_ESM.docx]

**Table S1** Schoenfeld residual test for the proportional hazards assumption of the Cox model

| Variable | X² | df | p |
| --- | --- | --- | --- |
| PA_group | 4.563 | 3 | 0.207 |
| Age | 1.974 | 1 | 0.160 |
| Gender | 3.159 | 1 | 0.076 |
| Marry | 0.233 | 1 | 0.630 |
| Education | 9.077 | 1 | 0.003 |
| Residence | 2.055 | 1 | 0.152 |
| Smoking | 3.508 | 1 | 0.061 |
| Drinking | 2.09 | 1 | 0.148 |
| GLOBAL | 18.012 | 10 | 0.055 |

PA_group: Physical activity group; X²: Chi-square statistic; df: Degrees of freedom; GLOBAL: Global test of the proportional hazards assumption for the entire model

**Table S2** Sensitivity analysis using 2015 cross-sectional data of CHARLS

|  | Model 1 | | Model 2 | | Model3 | |
| --- | --- | --- | --- | --- | --- | --- |
|  | OR(95% CI) | P | OR(95% CI) | P | OR(95% CI) | P |
| PA- | 1.00 (Reference) |  | 1.00 (Reference) |  | 1.00 (Reference) |  |
| Low | 0.935 (0.795–1.099) | 0.416 | 0.964 (0.816–1.138) | 0.665 | 0.961 (0.813–1.136) | 0.644 |
| Moderate | 0.735 (0.629–0.858) | <0.001 | 0.808 (0.689–0.948) | 0.009 | 0.812 (0.692–0.953) | 0.011 |
| High | 0.518 (0.425–0.631) | <0.001 | 0.618 (0.505–0.757) | <0.001 | 0.629 (0.513–0.770) | <0.001 |

PA: physical activity (PA- denotes the reference group); OR: odds ratio; CI: confidence interval

**Table S3** Sensitivity analysis of physical activity levels stratified by tertiles

|  | Model 1 | | Model 2 | | Model3 | |
| --- | --- | --- | --- | --- | --- | --- |
|  | HR(95% CI) | P | HR(95% CI) | P | HR(95% CI) | P |
| PA- | 1.00 (Reference) |  | 1.00 (Reference) |  | 1.00 (Reference) |  |
| Low | 0.864 (0.674–1.107) | 0.248 | 0.855 (0.666–1.097) | 0.218 | 0.852 (0.664–1.094) | 0.209 |
| Moderate | 0.744 (0.574–0.964) | 0.025 | 0.762 (0.587–0.989) | 0.041 | 0.763 (0.588–0.990) | 0.042 |
| High | 0.621 (0.478–0.807) | <0.001 | 0.690 (0.528–0.902) | 0.007 | 0.697 (0.533–0.911) | 0.008 |

PA: physical activity (PA- denotes the reference group); HR: hazard ratio; CI: confidence interval

**Table S4** Sensitivity analysis of physical activity levels using a combined low-activity group

|  | Model 1 | | Model 2 | | Model3 | |
| --- | --- | --- | --- | --- | --- | --- |
|  | HR(95% CI) | P | HR(95% CI) | P | HR(95% CI) | P |
| Low | 1.00 (Reference) |  | 1.00 (Reference) |  | 1.00 (Reference) |  |
| Moderate | 0.839 (0.710–0.991) | 0.038 | 0.876 (0.739–1.038) | 0.127 | 0.882 (0.744–1.046) | 0.148 |
| High | 0.693 (0.562–0.854) | 0.001 | 0.783 (0.628–0.976) | 0.030 | 0.793 (0.636–0.989) | 0.040 |

PA: physical activity (PA- denotes the reference group); HR: hazard ratio; CI: confidence interval
