# Supplementary figures and images for: The influence of physical activity on circadian syndrome: a nationwide prospective study based on the CHARLS cohort
Source: BMC Public Health. 2026 May 2;26:1909. doi: 10.1186/s12889-026-27627-3 (PMC13281635; doi:10.1186/s12889-026-27627-3)

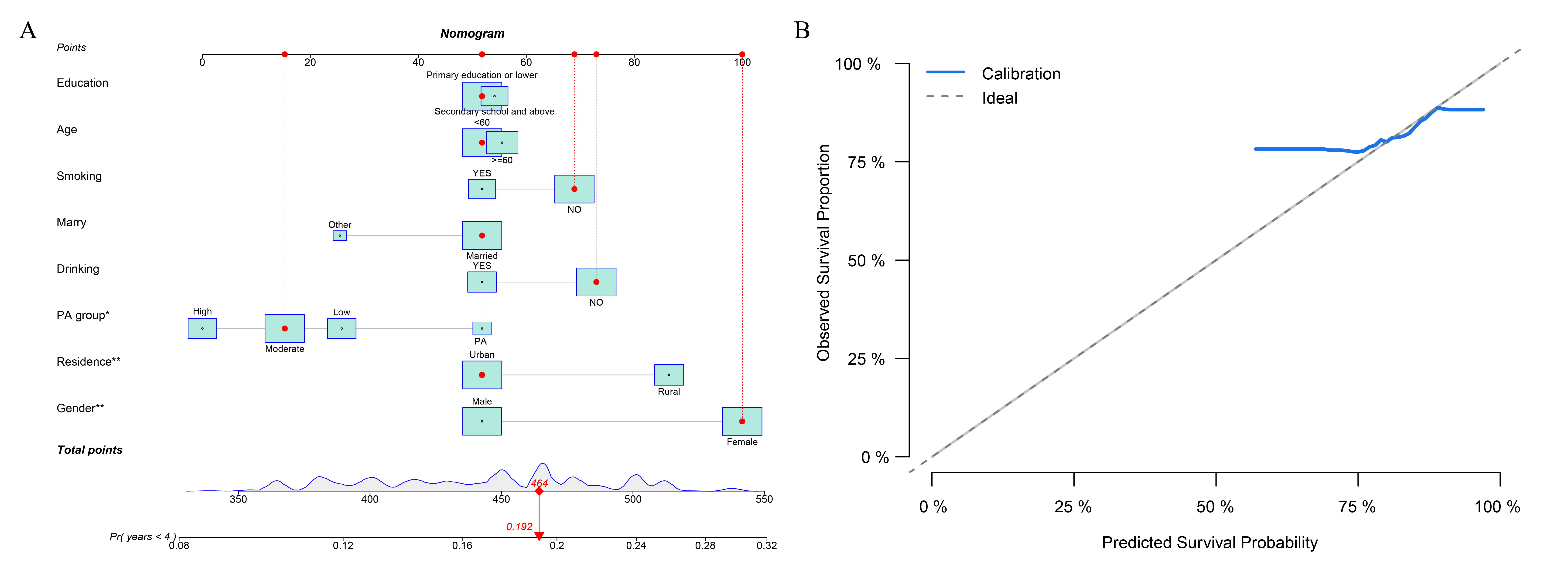

Supplement: Supplementary file 2 — Supplementary Material 2. Figure S1 Construction and validation of the nomogram. (A) The nomogram constructed based on covariates. (B) The calibration curve of the nomogram. [file 12889_2026_27627_MOESM2_ESM.tif]
